# Transplant conditioning with bortezomib, thalidomide, and melphalan and intensive 2 year post-transplant therapy for multiple myeloma in older patients

**DOI:** 10.1038/s41409-023-02119-9

**Published:** 2023-10-10

**Authors:** Christopher Strouse, Sarah L. Mott, Brian J. Smith, Margarida Magalhaes-Silverman, Umar Farooq, Fenghuang Zhan, Yogesh Jethava, Guido Tricot

**Affiliations:** 1 Division of Hematology, Oncology, and Blood & Marrow Transplantation, University of Iowa, Iowa City, IA USA; 2 Holden Comprehensive Cancer Center, University of Iowa, Iowa City, IA USA; 3 Winthrop P. Rockefeller Cancer Institute, University of Arkansas Medical Center, Little Rock, AK USA; 4 Indiana Blood & Marrow Transplant, Indianapolis, IN USA

**Keywords:** Myeloma, Stem-cell therapies, Quality of life, Combination drug therapy

## To the Editor:

Treatment with autologous stem cell transplant (ASCT) using melphalan 200 mg/m^2^ conditioning chemotherapy is a well-established and efficacious therapy for patients with multiple myeloma, even among eligible patients 65 years and older [1, 2]. However, despite improved outcomes, the predominant cause of death for patients remains their myeloma, highlighting the need to improve treatment efficacy for all patients [3]. This phase II study aimed to evaluate the tolerance, safety, and efficacy of an intensified conditioning with ASCT in patients ≥65 years of age with multiple myeloma.

## Methods

Transplant-eligible myeloma patients aged ≥65 with ≤12 months of prior therapy were enrolled. Prior transplant was not allowed. Patients with progressive disease on initial therapy were also excluded. The study was approved by the Institutional Review Board of the University of Iowa and performed in accordance with the principles of the Declaration of Helsinki.

Patients received one 4-day course of dexamethasone 40 mg/day, doxorubicin 10 mg/m^2^/day and a continuous infusion of cisplatin 10 mg/m^2^/day, cyclophosphamide 400 mg/m^2^/day, and etoposide 40 mg/m^2^/day (D-PACE). Pegfilgrastim was administered on d + 6 and d + 13 after the start of D-PACE and patients underwent apheresis to collect a target of >15 × 10^6^ CD34+ cells/kg. Patients then proceeded to transplant within 3 months of D-PACE. The conditioning regimen consisted of dexamethasone 20 mg PO days −4 to −1 and + 2 to + 5, bortezomib 1 mg/m^2^ IV days −4, −1, + 2, and + 5, thalidomide 100 mg PO days −4 to + 5, and melphalan 100 mg/m^2^ days −4 and −1 (70 mg/m^2^ days −4 and −1 if age >70), with autologous CD34+ cells on day 0. Maintenance therapy started as early as 4 weeks after transplant, and was delivered per institutional standard of care, allowing for substitutions in the case of toxicity. The institutional standard of care was considered 12 cycles of bortezomib, thalidomide and dexamethasone followed by 12 cycles of bortezomib, cyclophosphamide and dexamethasone, for a total fixed duration of 24 months. The most common substitution was lenalidomide for thalidomide in case of severe neuropathy.

The primary endpoint was progression free survival (PFS), measured from the start of protocol treatment until progression per International Myeloma Working Group consensus criteria or death. Overall response rate (ORR) and overall survival (OS) were secondary endpoints. Patients alive and disease-free at the off-study date were censored. All statistical testing of secondary endpoints was two-sided and assessed for significance at the 5% level.

## Results

There were 41 eligible patients enrolled between May 2013 and June 2017. The median age at enrollment was 68 years, and the oldest patient enrolled was 75 years old. Prior to enrollment, 26 patients had received prior therapy resulting in a stringent complete response (sCR) in 8%, complete response (CR) in 19%, very good partial response (VGPR) in 12%, partial response (PR) in 38%, and stable disease (SD) in 23% (Table 1).

**Table 1:** Baseline characteristics at study enrollment.

	*N* = 41
Age	
Median (range)	68 (65–75)
Sex	
Male	22 (54%)
Female	19 (46%)
Isotype	
IgG/IgA/LC/Others	26/6/8/1
Cytogenetic risk	
High^a^	17 (44%)
Standard	22 (56%)
Missing	2
Cytogenetic abnormalities	
Amplification 1q	2 (5%)
+1q	10 (26%)
−17p	3 (8%)
T[4;14]	0 (0%)
T[11;14]	6 (15%)
T[14;16]	2 (5%)
ISS	
Stage I	13 (35%)
Stage II	17 (46%)
Stage III	7 (19%)
Missing	4
Pre-protocol therapy	
No	15 (37%)
Yes	26 (63%)
Proteosome inhibitor	22 (85%)
IMiD	13 (50%)
Alkylator therapy	10 (38%)

^a^Defined as deletion of 17p, t(14;16), gain 1q (≥3 copies), or t(4;14) by fluorescence in situ hybridization (FISH) on CD-138 sorted plasma cells.

Two patients dropped out prior to transplant due to intolerance of D-PACE (*n* = 1) or failure of adequate CD34+ cell collection (*n* = 1). A median of 19.1 × 10^6^ CD34+ cells/kg (range: 11.3–73.8 × 10^6^ CD34+ cells/kg) were collected and a median of 9.8 × 10^6^ CD34+ cells/kg (range: 4.5–15.0 × 10^6^ CD34+ cells/kg) were infused with the transplant. Patients’ responses following transplant and prior to maintenance were sCR in 46%, CR in 8%, VGPR in 28%, PR in 15% and not evaluable (NE) in 3%.

**Fig. 1:**
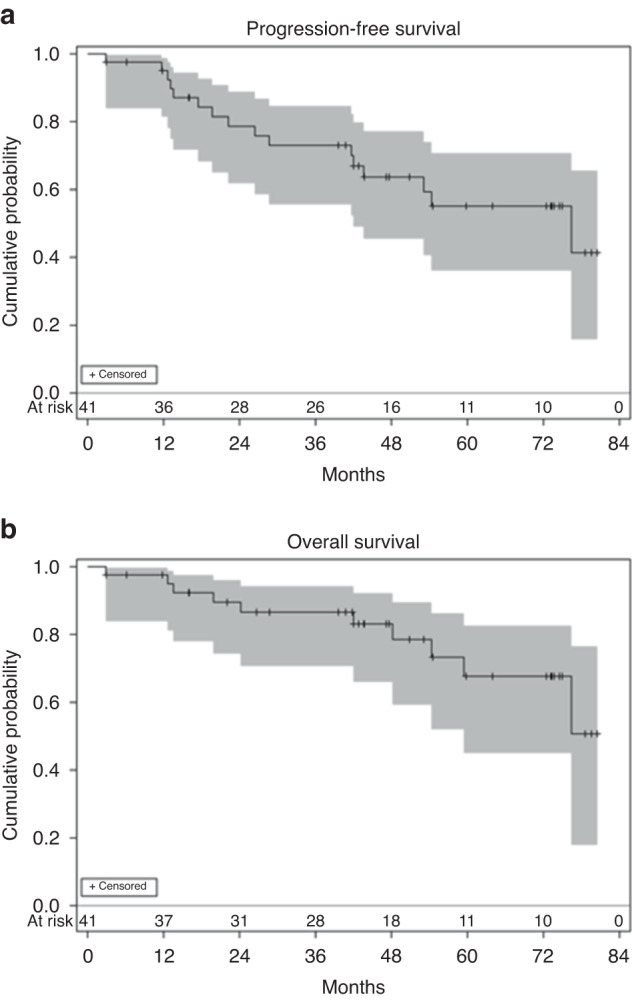
Efficacy Outcomes. **a** Progression free and (**b**) overall survival estimates and 95% confidence intervals.

Thirty-seven patients started maintenance therapy after a median of 34 days (range: 17–90 days). The median follow-up on study was 43.7 months (range: 2.9–80.5 months). The best response to therapy was sCr in 85%, VGPR in 7%, PR in 2%, and NE in 5%, with ORR of 95%. The estimated median PFS was 76.4 months (95% confidence interval [CI]: 42.0-not reached), and median OS was not reached. The PFS and OS at 48 months were 64% (95% CI: 45–77%) and 83% (95% CI: 66–92%), respectively (Fig. 1a, b). More than half of patients (54%) received all 24 cycles of maintenance therapy, 30% received 13–23 total cycles and 16% received 1–12 total cycles). For patients who completed the full 2-year course of therapy, the progression free survival and overall survival at 48 months were 81% (95% CI: 57–93%) and 100%, respectively.

Grade 3 or 4 hematologic toxicities were common. Median times to neutrophil and platelet engraftment were 11 days (range: 3–13) and 11 days (range: 3–30), respectively. Common grade 3 or 4 non-hematologic adverse events during the transplant and maintenance phases were infections (31% and 27%), diarrhea (21% and 11%), and electrolyte abnormalities such as hypophosphatemia (56% and 41%). Substitutions for thalidomide or bortezomib were made for neuropathy during maintenance therapy in 9 patients (24%). No patients had grade 3 or 4 peripheral neuropathy.

There was one transplant-related death within 100 days of transplant due to infectious complications.

## Discussion

The role of ASCT has increasingly been called into question, particularly among older patients [4]. Our study is the first to prospectively evaluate the safety and efficacy of ASCT in older patients with multiple myeloma using a novel agent conditioning regimen followed by an intensive 2-year maintenance therapy. The favorable best overall responses of sCR in 85% of patients and estimated median PFS of 76.4 months, reflect the feasibility, efficacy and safety of this intensive approach. Caution is warranted in extrapolating the results of this single-arm, single-institution study.

Prior reports of intensified conditioning regimens, combining alkylating agents, proteosome inhibitors, and/or immunomodulatory drugs (IMiDs), have reported rates of CR or better to be 22.1–38% [5–8], generally with acceptable safety parameters. In our study, the rate of CR or better prior to maintenance (46%) was comparable, and the rate of transplant-related mortality (2.6%) was similar to previously reported rates [9]. The use of intensive combination maintenance therapy also proved feasible in this study and was an important contributor to the prolonged median estimated PFS. Greater than 50% of patients were able to complete the 24-month maintenance regimen, which exceeds the 18 months median duration of lenalidomide only maintenance reported in the Myeloma XI study [10]. One important factor may have been the high stem cell dose used allowing subjects to start maintenance rapidly and preventing issues with cytopenias during maintenance. Other studies using intensive proteosome inhibitor/IMiD combination maintenance therapies have demonstrated their safety and have produced encouraging PFS. In the FORTE trial, patients randomized to the carfilzomib/lenalidomide maintenance arm had a 48-month PFS rate of 75%, while patients treated with carfilzomib/lenalidomide/dexamethasone maintenance in the ATLAS study had an estimated median PFS of 58 months [11, 12]. Our results add to this body of experience, which collectively demonstrates that even among this older population, intensified conditioning plus prolonged triplet therapy can be delivered safely and is associated with a high rate of deep responses and a prolonged PFS.

The 24-month defined duration of maintenance treatment, along with the long median PFS, resulted in a significant treatment-free period. This may be valuable to patients and to health care systems. Financial toxicity from myeloma therapy is common even among insured patients in the United States, and use of fixed-duration maintenance regimens, such as demonstrated in this study, could play a large role in attenuating this growing issue [13].

Based on these results, HSCT should remain a viable option for patients 65 and older and probably the therapy of choice if transplant-eligible.
